# Supplementary material for: Replication collisions induced by de-repressed S-phase transcription are connected with malignant transformation of adult stem cells
Source: Nat Commun. 2022 Nov 14;13:6907. doi: 10.1038/s41467-022-34577-y (PMC9663592; doi:10.1038/s41467-022-34577-y)
Supplement: Supplementary file 1 — Supplementary Info [file 41467_2022_34577_MOESM1_ESM.pdf]

Supplementary Information

Replication collisions induced by de-repressed S-phase transcription are connected with malignant transformation of adult stem cells

Ting Zhang[1], Carsten Künne[1,2], Dong Ding[1], Stefan Günther[1,3], Xinyue Guo[1], Yonggang Zhou[1], Xuejun Yuan[*,1] and Thomas Braun[*, 1, 4, 5]

[1]Department of Cardiac Development and Remodeling, Max-Planck-Institute for Heart and Lung Research, Bad Nauheim, Germany

[2]Bioinformatics Core Unit (BCU), Max Planck Institute for Heart and Lung Research, Bad Nauheim, Germany

[3]CPI Deep Sequencing Platform, Max Planck Institute for Heart and Lung Research, Bad Nauheim, Germany

[4]German Center for Cardiovascular Research (DZHK), Rhine Main, Germany

[5]German Center for Lung Research (DZL), Giessen, Germany

*Corresponding authors: Xuejun Yuan and Thomas Braun; xuejun.yuan@mpi-bn.mpg.de, thomas.braun@mpi-bn.mpg.de

**Supplementary Information**	**Page**
Supplementary Figure 1	3
Supplementary Figure 2	5
Supplementary Figure 3	7
Supplementary Figure 4	9
Supplementary Figure 5	11
Supplementary Figure 6	13
Supplementary Figure 7	15
Supplementary Figure 8	17
Supplementary Figure 9	19
Supplementary Figure 10	21

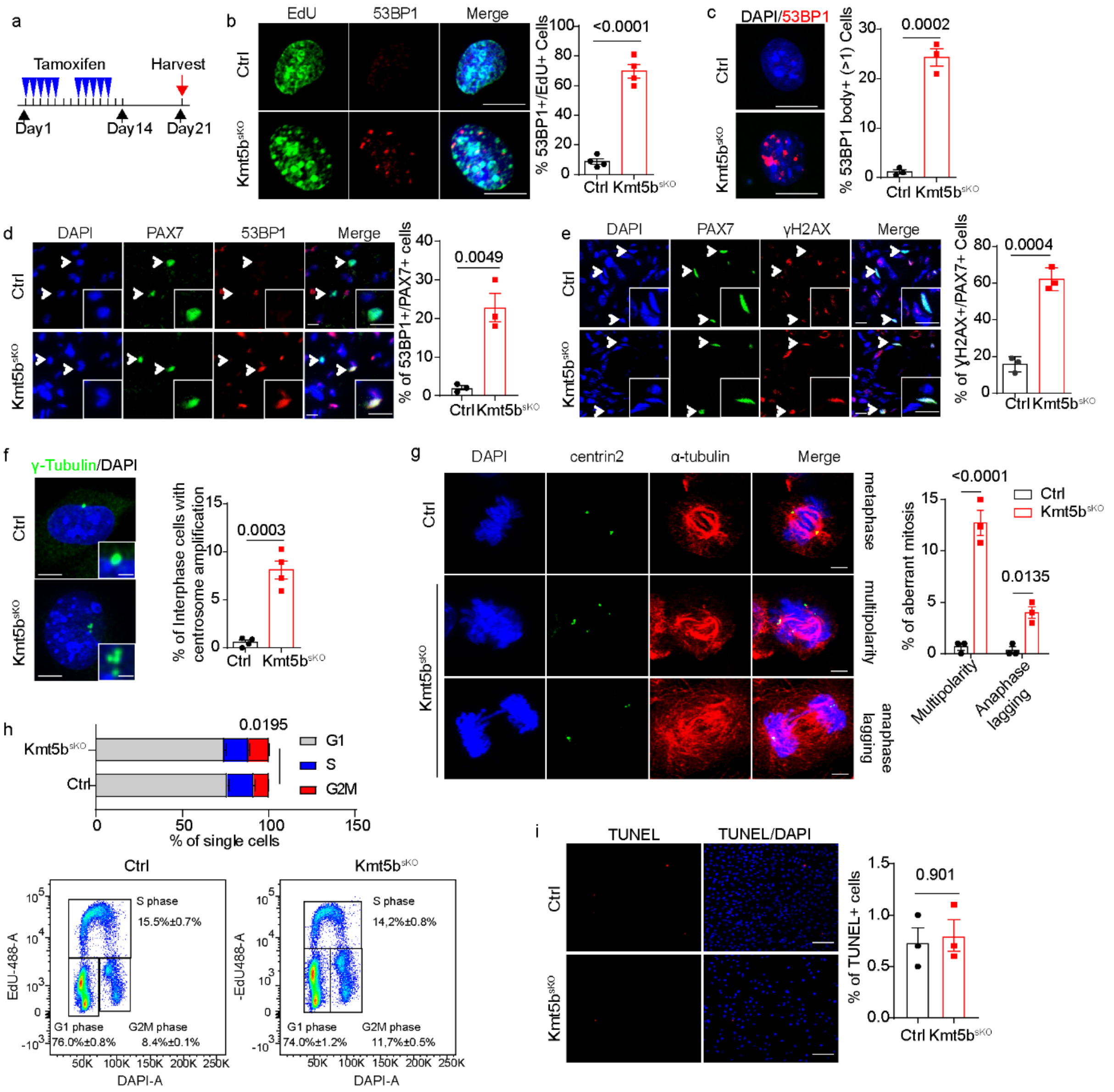

Supplementary Figure 1: *Kmt5b* prevents aberrant mitosis and genomic instability in proliferating MuSC.

(a) Schematic description of tamoxifen administration in *Ctrl* and *Kmt5b^{sKO}* mice. Mice were analyzed at least 7 days after the last tamoxifen injection.

(b) Immunofluorescence staining for EdU and 53BP1 in *Ctrl* and *Kmt5b^{sKO}* MuSCs after 4 days in culture (n=4 mice). DNA was stained by DAPI. Scale bar: 10 µm. Quantification of 53BP1^{+}/EdU^{+} is on the right.

(c) Images of 53BP1 nuclear bodies in nuclei of *Ctrl* and *Kmt5b^{sKO}* MuSCs in G1 phase (EdU^{-} with 2N DNA content based on DAPI intensity) (n=3 mice). DNA was stained by DAPI. Scale bar: 10 µm. Quantification of G1-phase MuSCs with >1 53BP1^{+} nuclear bodies is on the right.

(d) &(e) Immunofluorescence staining for (d) 53BP1 and (e) γH2AX in Pax7^{+} MuSCs (indicated by arrowheads) in TA muscles of *Ctrl* and *Kmt5b^{sKO}* mice, 7-days after CTX injections. Scale bar: 10 µm. Quantification of (d) 53BP1^{+}/PAX7^{+} and (e) γH2AX^{+}/PAX7^{+} MuSCs detected in 10 sections from each animal in the right panel (n=3 mice).

(f) Immunofluorescence analysis of γ-tubulin demonstrating amplified centrosome in *Kmt5b*^{sKO} MuSCs. Scale bar: 5 µm. Scale bars in the magnified images represent 1 µm. Quantification of interphase MuSCs with amplified centrosome (>2) in *Ctrl* and *Kmt5b*^{sKO} MuSCs (n=4 mice) is on the right.
(g) Immunofluorescence staining for centrin2 and α-tubulin revealing multipolar metaphases and bridging anaphases in *Kmt5b*^{sKO} MuSCs in comparison to *Ctrl* MuSCs after 4 days in growth medium (n=3 mice). Scale bar: 2 µm. Quantification of cells with aberrant mitosis is on the right.
(h) Flow cytometry-based cell cycle analysis by EdU/DAPI staining in *Ctrl* and *Kmt5b*^{sKO} MuSCs after 4 days in culture (n=3 mice). Quantification of percentages of cells in different cell cycle phase is shown in the upper panel.
(i) TUNEL assay of 4-days cultured *Ctrl* and *Kmt5b*^{sKO} MuSCs (n=3 mice). Scale bar: 100 µm Quantification of TUNEL^{+} cells is on the right.
Data are shown as mean ± SEM. Two-sided p-values for unpaired Student's t-tests are indicated in the figure. Source data are provided in the Source Data file.

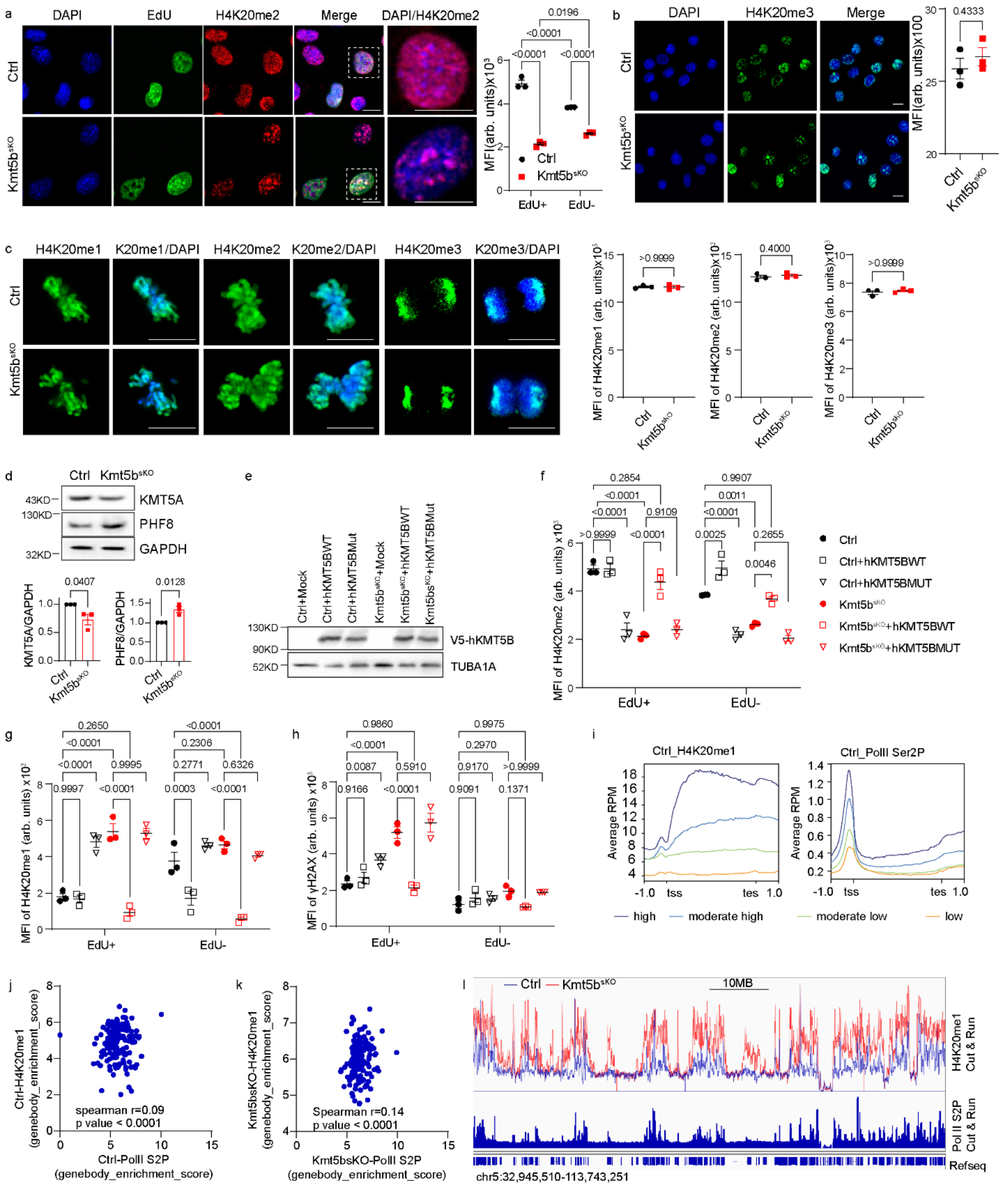

Supplementary Figure 2: Loss of *Kmt5b* alters the extent of H4K20 methylation in MuSC. (a) Immunofluorescence analysis of EdU incorporation and H4K20me2 level in *Ctrl* and *Kmt5b^{sKO}* MuSCs. DNA was stained by DAPI. Magnified images of DAPI/H4K20me2 staining in selected EdU^{+} MuSCs are shown. Scale bar: 10 µm. Median of fluorescence intensity (MFI, arbitrary units (arb. unitsarb. units)) of H4K20me2 in EdU^{+}/EdU^{-} cells is shown on the right (n=3 mice). Two-way ANOVA.

(b) Immunofluorescence analysis of H4K20me3 in *Ctrl* and *Kmt5b*^{sKO} MuSCs after 4-days in culture. DNA was stained by DAPI. Scale bar: 10 µm. Median of fluorescence intensity (MFI, arbitrary units (arb. units)) of H4K20me3 is shown on the right (n=3 mice). Unpaired two-sided Student's t-test. (c) Immunofluorescence analysis of H4K20me1, H4K20me2, and H4K20me3 in *Ctrl* and *Kmt5b*^{sKO} MuSCs during metaphase. DNA was stained by DAPI. Scale bars: 10 µm. Quantification of MFI is shown on the right (n=3 mice). Unpaired two-sided Student's t-test. (d) Western blot analysis of KMT5A and PHF8 in *Ctrl and Kmt5b*^{sKO} MuSCs (n=3 mice). GAPDH was used as internal control. Unpaired two-sided Student's t-test.

(e) Western blot analysis of overexpressed wildtype (WT) and catalytically inactive (MUT) hKMT5B with V5 tag in *Ctrl and Kmt5b*^{sKO} MuSCs (n=3 mice). TUBA1A served as the loading control.

(f-h) Immunofluorescence analysis (MFI) of (f) H4K20me2, (g) H4K20me1, and (h) γH2AX in EdU^{+}/EdU^{-} *Ctrl and Kmt5b*^{sKO} MuSCs after transduction with empty, wildtype (WT) and catalytically-inactive (MUT) hKMT5B vectors (n=3 mice). Two-way ANOVA.

(i) Average levels of H4K20me1 (left panel) and Pol II S2P (right panel) in four groups of genes by abundance of H4K20me1 CUT&RUN signals within gene bodies of all coding genes in proliferating *Ctrl* MuSC.

(j, k) Enrichment of H4K20me1 and Pol II S2P binding on bodies of genes coding for small non-coding RNAs, including rRNAs, snRNAs, snoRNAs, and scaRNAs in *Ctrl* (j) and *Kmt5b*^{sKO} (k) MuSCs. Spearman's correlation coefficient r-values are indicated. Two-sided P-values indicate the significance of the correlation determined by r values and sample size.

(l) IGV snapshots of a representative region on mouse chromosome chr5, showing CUT&RUN signals for H4K20me1 in *Ctrl* and *Kmt5b*^{sKO} MuSCs (upper panel) and Pol II S2P in *Ctrl* MuSCs (lower panel).

Data are shown as mean ± SEM. P values are indicated in the figure. Source data are provided in the Source Data file.

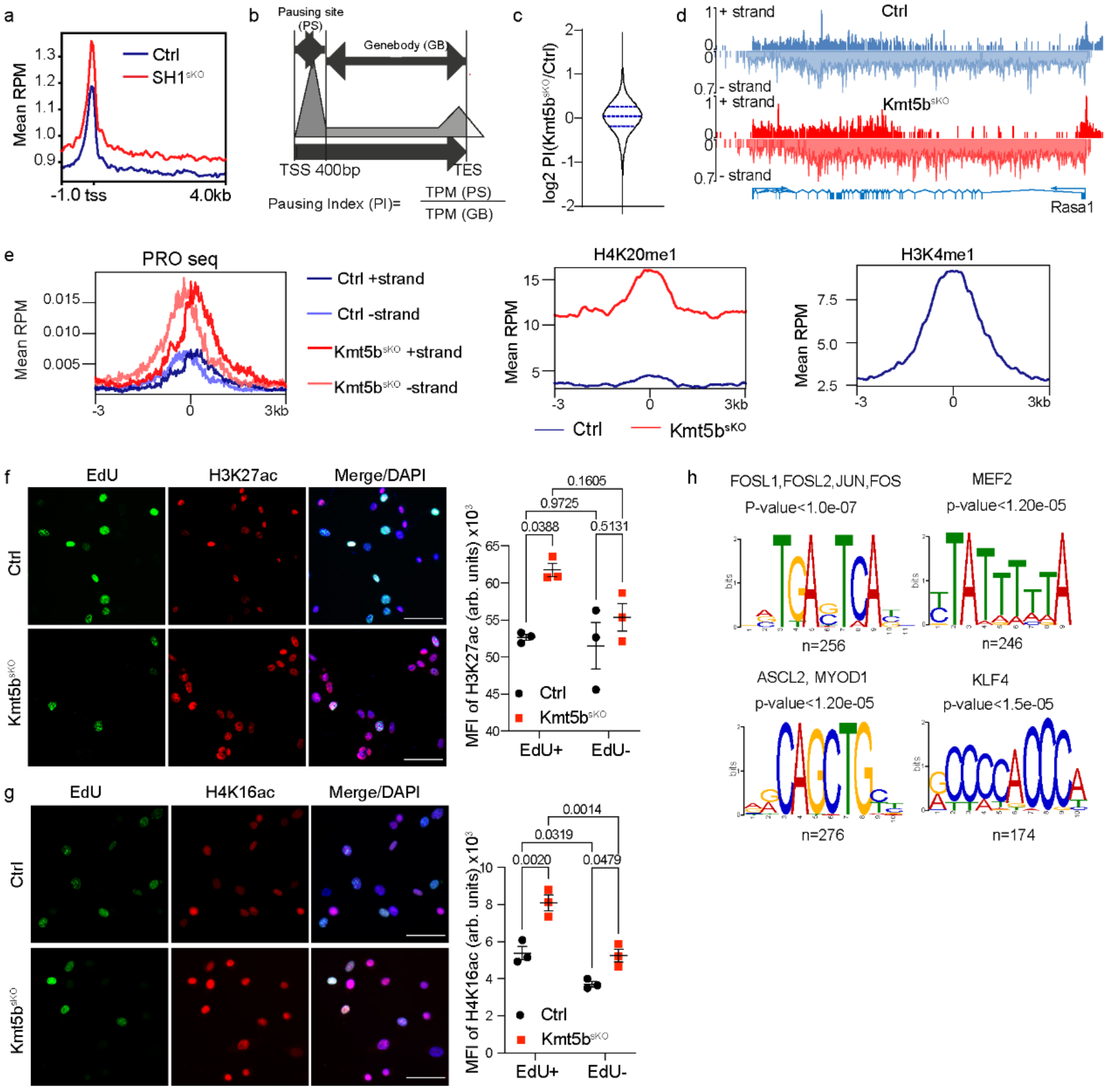

Supplementary Figure 3: PRO-seq analysis of *Ctrl* and *Kmt5b^{sKO}* MuSCs and identification of transcription factor binding sites in enhancer regions showing increased eRNA transcription.

(a) Average profiles of PRO-seq at the TSS and 4kb downstream of 8020 genes with increased accumulation of H4K20me1 and Pol II S2P. TSS: transcription start site

(b) Illustration of the calculation for the polymerase II pausing index using the ratios of densities of PRO-seq signals at pausing sites (TSS to TSS +500bp) vs gene bodies (TSS +500bp to TES). TSS: transcription start site; TES: transcription termination site.

(c) Violin plot of the log2 pausing index (*Kmt5b^{sKO}*/*Ctrl*). The blue lines indicate the upper/lower adjacent values defining third/first quartiles and the median.

(d) Genome browser track of PRO-seq signals in the *RaS1* gene locus in *Ctrl* and *Kmt5b^{sKO}* MuSCs. Positive and negative values on the y-axis represent library-normalized reads mapping to the plus and minus strands, respectively.

(e) Average profiles of PRO-seq, H4K20me1 CUT&RUN around enhancer regions (±3kb from the center) in *Ctrl* and *Kmt5b^{sKO}* MuSCs. H3K4me1 ChIP-seq signals (BioProject: PRJNA412267) were used to identify enhancer regions.

(f, g) Immunofluorescence analysis of (f) H3K27ac and (g) H4K16ac in EdU^+/EdU^- *Ctrl* and $Kmt5b^{sKO}$ MuSCs after 4-days in culture. DNA was stained by DAPI. Scale bar: 50 µm. Median of fluorescence intensity (MFI, arbitrary units (arb. units)) of (f) H3K27ac and (g) H4K16ac in EdU^+/EdU^- cells is shown on the right (n=3 mice). Data are shown as mean ± SEM. Two-way ANOVA.
(h) Identification of enriched transcription factor binding motifs by MEME and DMEME within the center of enhancers regions with increased eRNA transcription after loss of *Kmt5b*. Corresponding transcription factors were identified by TOMTOM. Source data are provided in the Source Data file.

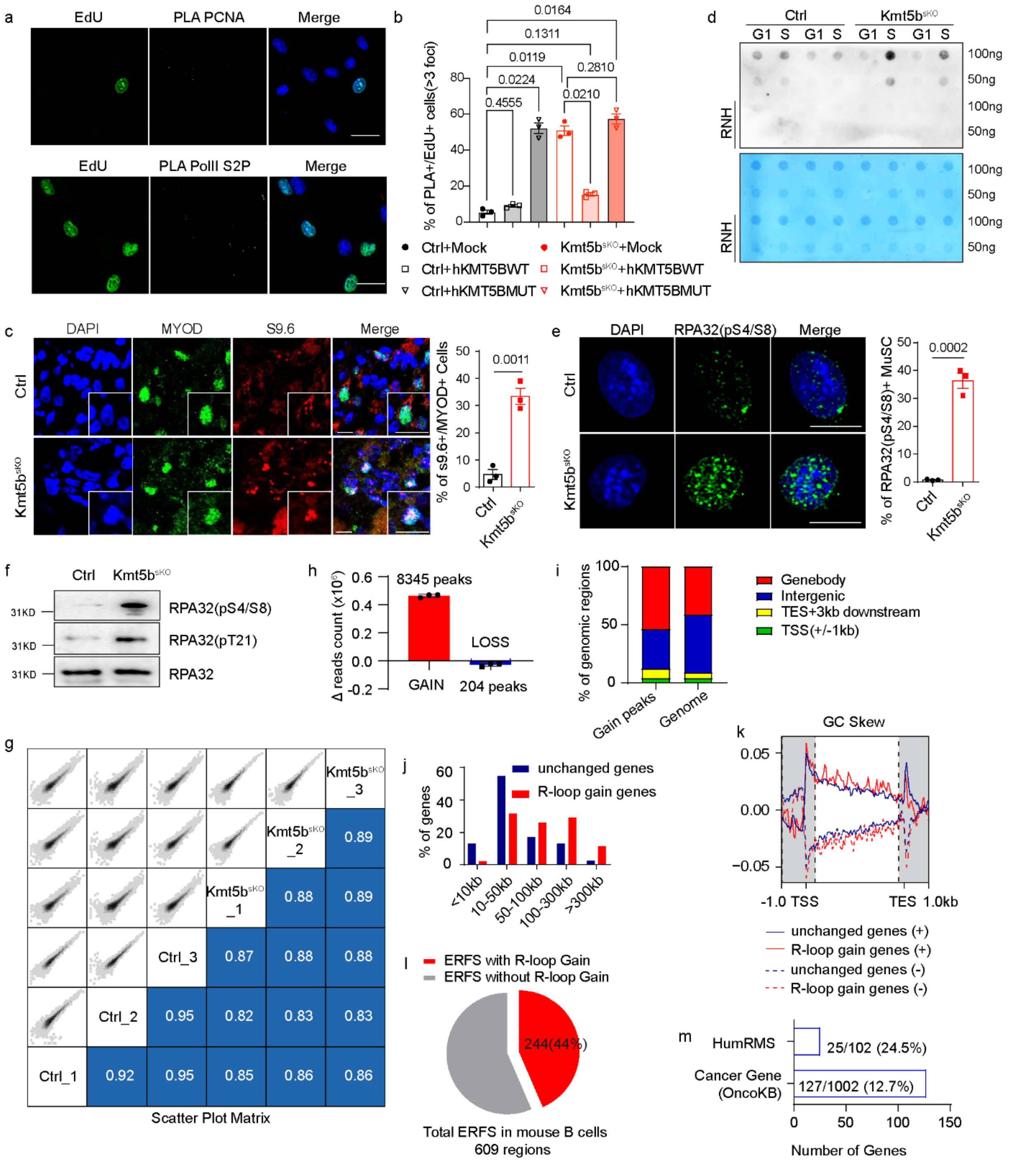

Supplementary Figure 4: Increased R-loop formation and DNA damage responses in MuSCs after loss of *Kmt5b*.

(a) Representative images of controls (PCNA or PolII S2P) using a single antibody in the PLA assay together with EdU labeling. Scale bar: 20 µm.

(b) PLA of PCNA and PolII S2P in EdU^{+} *Ctrl* and *Kmt5b^{sKO}* MuSCs after transduction of empty vector, wildtype (WT) and catalytically-inactive (MUT) h*KMT5B* (n=3 mice). Data represent mean ± SEM. One-way ANOVA.

(c) Immunofluorescence staining for MYOD and S9.6 in MuSCs from regenerating TA muscles of *Ctrl* and Kmt5b^{sKO} mice, 7 days after CTX injection (n=3 mice). DNA was stained by DAPI. Scale bar: 20 µm. Quantification of S9.6^{+}/MYOD^{+} cells is shown on the right. Data represent mean ± SEM. Unpaired two-sided Student's t-test.

(d) Dot blot of R-loops in sorted G1 and S-phase cells from *Ctrl* and *Kmt5b^{sKO}* MuSCs.

(e) Immunofluorescence analysis of RPA32 pS4/S8 in *Ctrl* and *Kmt5b^{sKO}* MuSCs after 4 days in culture (n=3 mice). Quantification of RPA32 pS4/S8^{+} MuSCs is on the right. Scale bar: 10 µm. Data represent mean ± SEM. Unpaired two-sided Student's t-test.

(f) Western blot analysis of RPA32 pS4/S8 and RPA32 pT21 in *Ctrl* and Kmt5b^{sKO} MuSCs after 4-days in culture. Total RPA32 served as internal control (n=2 mice).

(g) Spearman rank correlations between pairwise comparisons of DRIP-seq peaks from replicates. The upper-left section of the diagram provides smoothened scatter plot data whereas the lower-right section displays rank correlation values.

(h) Relative differences of uniquely mapped reads between increased (5134) and decreased (210) R-loop peaks when comparing *Ctrl* and *Kmt5b^{sKO}* MuSC . Newly acquired R-loops dominate over losses (n=3 mice). Data represent mean ± SEM.

(i) Distribution of newly acquired DRIP-seq peaks in percentages across several genomic regions after *Kmt5b* inactivation.

(j) Distribution of gene length of 1508 coding genes with newly acquired R-loops and enrichment of H4K20me1 and Pol II S2P in comparison with coding genes without changes in R-loop formation.

(k) GC skews over gene bodies of genes with newly acquired R-loops and enrichment of H4K20me1 and Pol II S2P in comparison with coding genes without changes in R-loop formation.

(l) Pie chart showing the ratio of fragile early replication sites (ERFS) with newly acquired R-loops. The number of ERFS determined in B-cells is indicated.

(m) Representation of genes with newly acquired R-loops corresponding to orthologous mutated cancer genes annotated by OncoKB in human rhabdomyosarcomas. Percentages for each category are indicated. Source data are provided in the Source Data file.

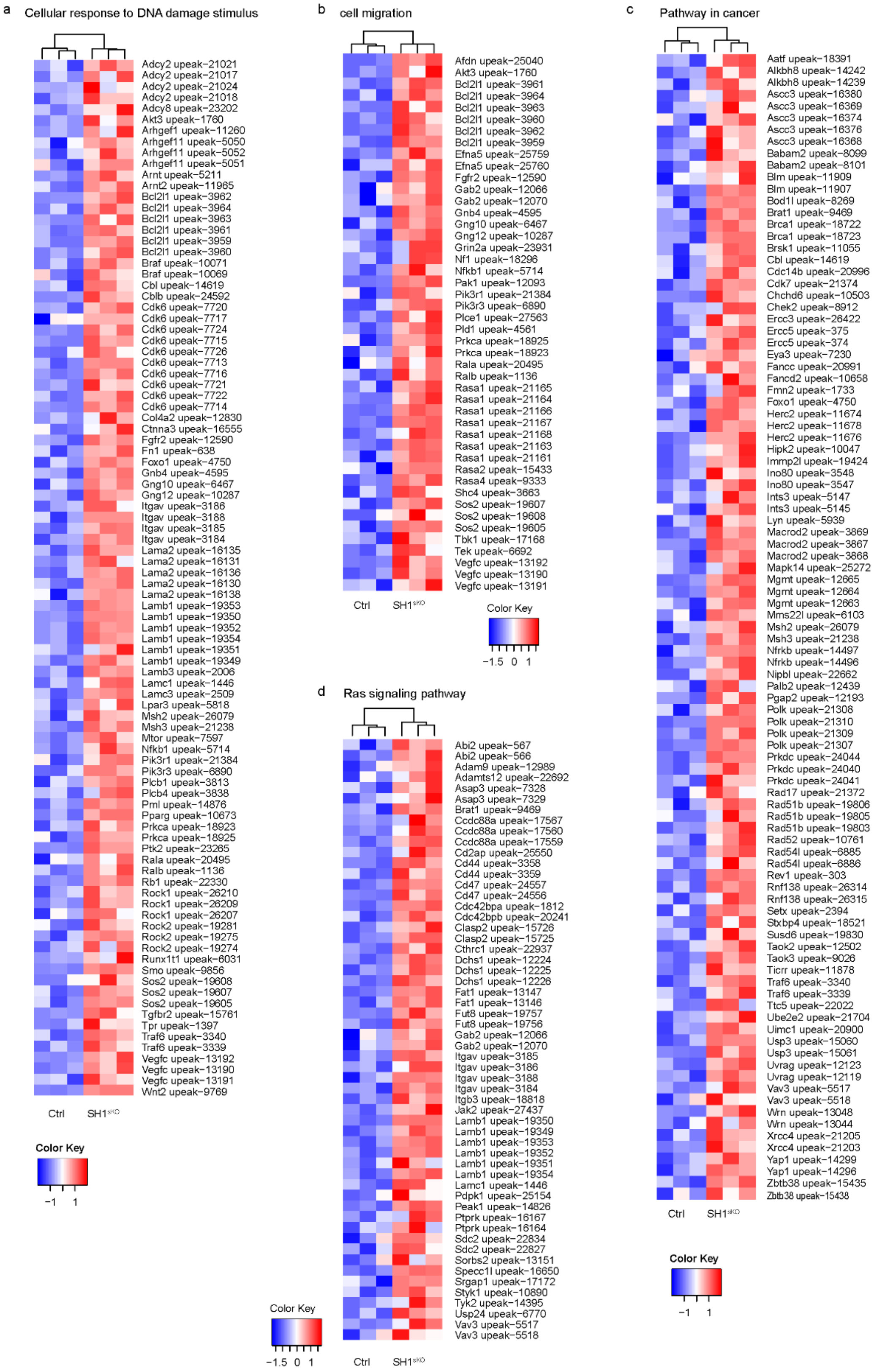
a
Cellular response to DNA damage stimulus
Adcy2 upeak−21021
Adcy2 upeak−21017
Adcy2 upeak−21024
Adcy2 upeak−21018
Adcy8 upeak−23202
Akt3 upeak−1760
Arhgef1 upeak−11260
Arhgef11 upeak−5050
Arhgef11 upeak−5052
Arhgef11 upeak−5051
Arnt upeak−5211
Arnt2 upeak−11965
Bcl2l1 upeak−3962
Bcl2l1 upeak−3964
Bcl2l1 upeak−3963
Bcl2l1 upeak−3961
Bcl2l1 upeak−3959
Bcl2l1 upeak−3960
Braf upeak−10071
Braf upeak−10069
Cbl upeak−14619
Cblb upeak−24592
Cdk6 upeak−7720
Cdk6 upeak−7717
Cdk6 upeak−7724
Cdk6 upeak−7715
Cdk6 upeak−7726
Cdk6 upeak−7713
Cdk6 upeak−7716
Cdk6 upeak−7721
Cdk6 upeak−7722
Cdk6 upeak−7714
Col4a2 upeak−12830
Ctnna3 upeak−16555
Fgfr2 upeak−12590
Fn1 upeak−638
Foxo1 upeak−4750
Gnb4 upeak−4595
Gng10 upeak−6467
Gng12 upeak−10287
Itgav upeak−3186
Itgav upeak−3188
Itgav upeak−3185
Itgav upeak−3184
Lama2 upeak−16135
Lama2 upeak−16131
Lama2 upeak−16136
Lama2 upeak−16130
Lama2 upeak−16138
Lamb1 upeak−19353
Lamb1 upeak−19350
Lamb1 upeak−19352
Lamb1 upeak−19354
Lamb1 upeak−19351
Lamb1 upeak−19349
Lamb3 upeak−2006
Lamc1 upeak−1446
Lamc3 upeak−2509
Lpar3 upeak−5818
Msh2 upeak−26079
Msh3 upeak−21238
Mtor upeak−7597
Nfkb1 upeak−5714
Pik3r1 upeak−21384
Pik3r3 upeak−6890
Plcb1 upeak−3813
Plcb4 upeak−3838
Pml upeak−14876
Pparg upeak−10673
Prkca upeak−18923
Prkca upeak−18925
Ptk2 upeak−23265
Rala upeak−20495
Ralb upeak−1136
Rb1 upeak−22330
Rock1 upeak−26210
Rock1 upeak−26209
Rock1 upeak−26207
Rock2 upeak−19281
Rock2 upeak−19275
Rock2 upeak−19274
Runx1t1 upeak−6031
Smo upeak−9856
Sos2 upeak−19608
Sos2 upeak−19607
Sos2 upeak−19605
Tgfbr2 upeak−15761
Tpr upeak−1397
Traf6 upeak−3340
Traf6 upeak−3339
Vegfc upeak−13192
Vegfc upeak−13190
Vegfc upeak−13191
Wnt2 upeak−9769
Ctrl
SH1sKO
Color Key
−1 1
b
cell migration
Afdn upeak−25040
Akt3 upeak−1760
Bcl2l1 upeak−3961
Bcl2l1 upeak−3964
Bcl2l1 upeak−3963
Bcl2l1 upeak−3960
Bcl2l1 upeak−3962
Bcl2l1 upeak−3959
Efna5 upeak−25759
Efna5 upeak−25760
Fgfr2 upeak−12590
Gab2 upeak−12066
Gab2 upeak−12070
Gnb4 upeak−4595
Gng10 upeak−6467
Gng12 upeak−10287
Grin2a upeak−23931
Nf1 upeak−18298
Nfkb1 upeak−5714
Pak1 upeak−12093
Pik3r1 upeak−21384
Pik3r3 upeak−6890
Plce1 upeak−27563
Pld1 upeak−4561
Prkca upeak−18925
Prkca upeak−18923
Rala upeak−20495
Ralb upeak−1136
Rasa1 upeak−21165
Rasa1 upeak−21164
Rasa1 upeak−21166
Rasa1 upeak−21167
Rasa1 upeak−21168
Rasa1 upeak−21163
Rasa1 upeak−21161
Rasa2 upeak−15433
Rasa4 upeak−9333
Shc4 upeak−3663
Sos2 upeak−19607
Sos2 upeak−19608
Sos2 upeak−19605
Tbk1 upeak−17168
Tek upeak−6692
Vegfc upeak−13192
Vegfc upeak−13190
Vegfc upeak−13191
Ctrl
SH1sKO
Color Key
−1.5 0 1
c
Pathway in cancer
Aatf upeak−18391
Alkbh8 upeak−14242
Alkbh8 upeak−14239
Ascc3 upeak−16380
Ascc3 upeak−16369
Ascc3 upeak−16374
Ascc3 upeak−16376
Ascc3 upeak−16368
Babam2 upeak−8099
Babam2 upeak−8101
Blm upeak−11909
Blm upeak−11907
Bod1l upeak−8269
Brat1 upeak−9469
Brca1 upeak−18722
Brca1 upeak−18723
Brsk1 upeak−11055
Cbl upeak−14619
Cdc14b upeak−20996
Cdk7 upeak−21374
Chchd6 upeak−10503
Chek2 upeak−8912
Ercc3 upeak−26422
Ercc5 upeak−375
Ercc5 upeak−374
Eya3 upeak−7230
Fancc upeak−20991
Fancd2 upeak−10658
Fmn2 upeak−1733
Foxo1 upeak−4750
Herc2 upeak−11674
Herc2 upeak−11678
Herc2 upeak−11676
Hipk2 upeak−10047
Immp2l upeak−19424
Ino80 upeak−3548
Ino80 upeak−3547
Ints3 upeak−5147
Ints3 upeak−5145
Lyn upeak−5939
Macrod2 upeak−3869
Macrod2 upeak−3867
Macrod2 upeak−3868
Mapk14 upeak−25272
Mgmt upeak−12665
Mgmt upeak−12664
Mgmt upeak−12663
Mms22l upeak−6103
Msh2 upeak−26079
Msh3 upeak−21238
Nfrkb upeak−14497
Nfrkb upeak−14496
Nipbl upeak−22662
Palb2 upeak−12439
Pgap2 upeak−12193
Polk upeak−21308
Polk upeak−21310
Polk upeak−21309
Polk upeak−21307
Prkdc upeak−24044
Prkdc upeak−24040
Prkdc upeak−24041
Rad17 upeak−21372
Rad51b upeak−19806
Rad51b upeak−19805
Rad51b upeak−19803
Rad52 upeak−10761
Rad54l upeak−6885
Rad54l upeak−6886
Rev1 upeak−303
Rnf138 upeak−26314
Rnf138 upeak−26315
Setx upeak−2394
Stxbp4 upeak−18521
Susd6 upeak−19830
Taok2 upeak−12502
Taok3 upeak−9026
Ticrr upeak−11878
Traf6 upeak−3340
Traf6 upeak−3339
Ttc5 upeak−22022
Ube2e2 upeak−21704
Uimc1 upeak−20900
Usp3 upeak−15060
Usp3 upeak−15061
Uvrag upeak−12123
Uvrag upeak−12119
Vav3 upeak−5517
Vav3 upeak−5518
Wrn upeak−13048
Wrn upeak−13044
Xrcc4 upeak−21205
Xrcc4 upeak−21203
Yap1 upeak−14299
Yap1 upeak−14296
Zbtb38 upeak−15435
Zbtb38 upeak−15438
Ctrl
SH1sKO
Color Key
−1 1
d
Ras signaling pathway
Abi2 upeak−567
Abi2 upeak−566
Adam9 upeak−12989
Adamts12 upeak−22692
Asap3 upeak−7328
Asap3 upeak−7329
Brat1 upeak−9469
Cccdc88a upeak−17567
Cccdc88a upeak−17560
Cccdc88a upeak−17559
Cd2ap upeak−25550
Cd44 upeak−3358
Cd44 upeak−3359
Cd47 upeak−24557
Cd47 upeak−24556
Cdc42bpa upeak−1812
Cdc42bpb upeak−20241
Clasp2 upeak−15726
Clasp2 upeak−15725
Cthrc1 upeak−22937
Dchs1 upeak−12224
Dchs1 upeak−12225
Dchs1 upeak−12226
Fat1 upeak−13147
Fat1 upeak−13146
Fut8 upeak−19757
Fut8 upeak−19756
Gab2 upeak−12066
Gab2 upeak−12070
Itgav upeak−3185
Itgav upeak−3186
Itgav upeak−3188
Itgav upeak−3184
Itgb3 upeak−18818
Jak2 upeak−27437
Lamb1 upeak−19350
Lamb1 upeak−19349
Lamb1 upeak−19353
Lamb1 upeak−19352
Lamb1 upeak−19351
Lamb1 upeak−19354
Lamc1 upeak−1446
Pdpk1 upeak−25154
Peak1 upeak−14826
Ptprk upeak−16167
Ptprk upeak−16164
Sdc2 upeak−22834
Sdc2 upeak−22827
Sorbs2 upeak−13151
Specc1l upeak−16650
Srgap1 upeak−17172
Styk1 upeak−10890
Tyk2 upeak−14395
Usp24 upeak−6770
Vav3 upeak−5517
Vav3 upeak−5518
Color Key
−1.5 0 1
Ctrl
SH1sKO

Supplementary Figure 5: Heatmaps of cancer-related genes with newly acquired R-loops. (a-d) Gene ontology and KEGG pathway analysis of cancer-related genes with newly acquired R-loops. Genes with newly acquired R-loops fall in different categories including cellular response to DNA damage response (a), cell migration (b), pathway in cancer (c), and RAS signaling (d). The color key in the heatmaps reflects the frequency of R-loop formation.

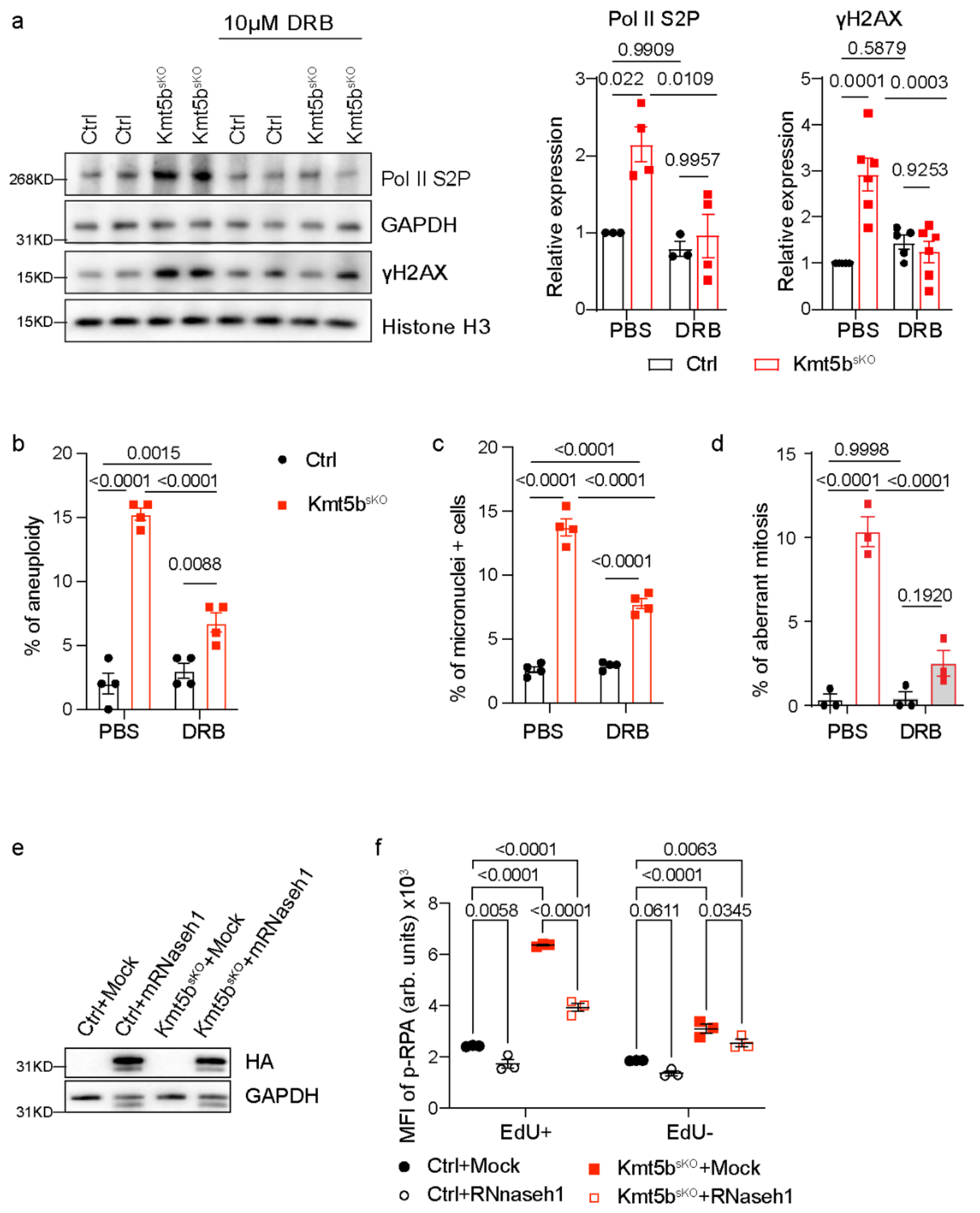

Supplementary Figure 6: Inhibition of S-phase transcription ameliorates aberrant mitosis and genome instability

(a) Western blot analysis of Pol II S2P and γH2AX in mock or DRB treated *Ctrl* and *Kmt5b*^{sKO} MuSCs. GAPDH and Histone H3 served as loading controls. Band intensities were quantified and normalized values are on the right (PolII S2P *Ctrl* n=3 mice, *Kmt5b*^{sKO} n=4 mice; γH2AX Ctrl n=5 mice; *Kmt5b*^{sKO} n=6 mice).

(b, c, d) Percentages of (b) cells with aneuploidy (n=4 mice), (c) micronuclei (n=4 mice) and (d) aberrant mitosis (n=3 mice) in mock and DRB-treated *Ctrl* and *Kmt5b*^{sKO} MuSCs.

(e) Western blot analysis of transduced HA-tagged mouse RNaseh1 in *Ctrl and Kmt5b*^{sKO} MuSCs. GAPDH served as endogenous control (n=3 mice).
(f) Immunofluorescence analysis (MFI) of p-RPA (S33) in EdU^{+}/EdU^{-} *Ctrl and Kmt5b*^{sKO} MuSCs with or without transduction of mouse *RNaseh1* (n=3 mice).
Data are shown as mean ± SEM. Two-way ANOVA. Source data are provided in the Source Data file.

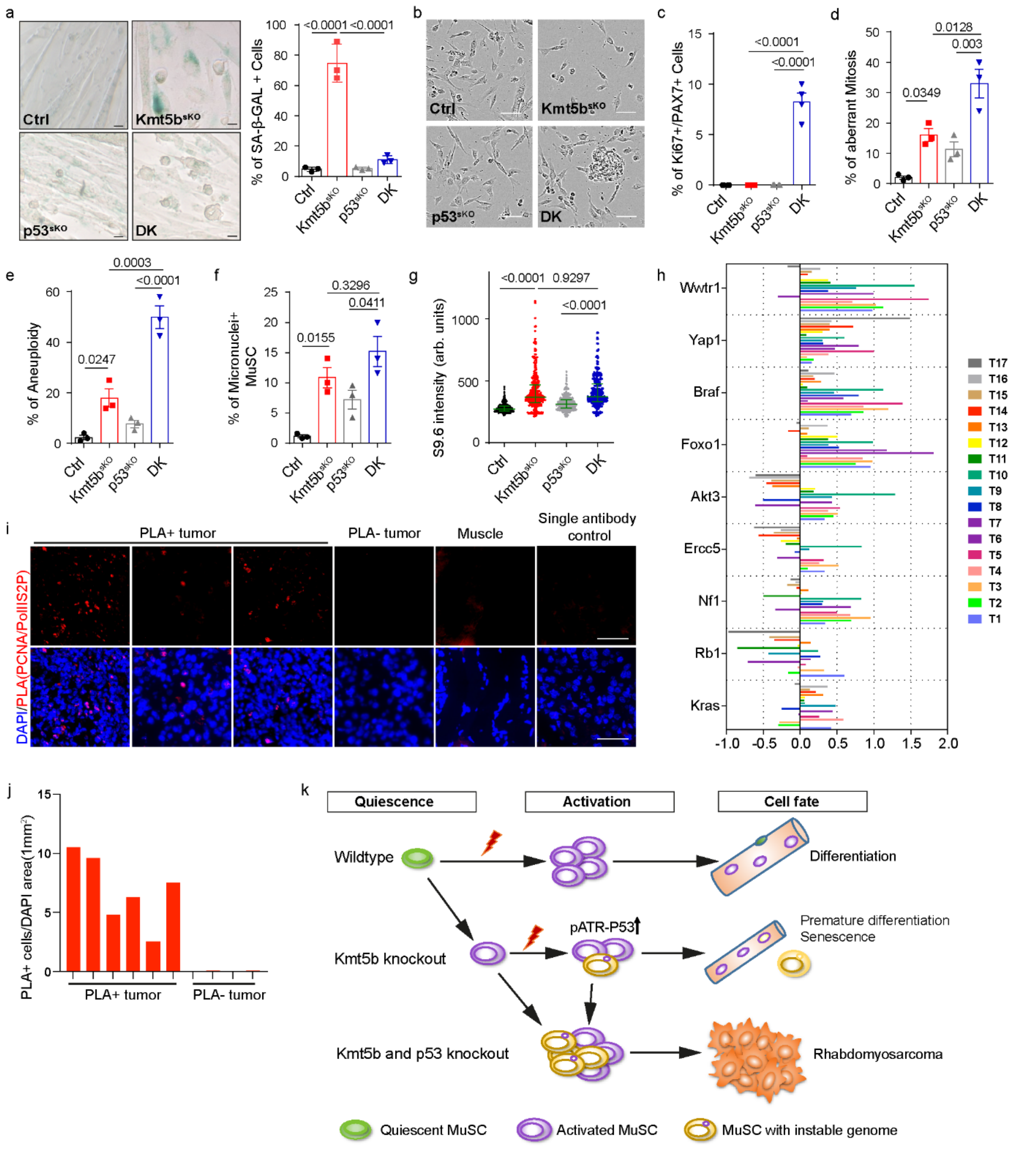

Supplementary Figure 7 Inactivation of *p53* in *Kmt5b^{sKO}* MuSCs prevents cellular senescence and enhances genomic instability.

(a) SA-β-GAL staining of cultured *Ctrl*, *Kmt5b^{sKO}*, *p53^{sKO}*, and *DK* MuSCs (n=3 mice). Quantification of SA-β-GAL positive cells is on the right. Scale bar: 10 µm.

(b) Representative bright field images of *Ctrl*, *Kmt5b^{sKO}*, *p53^{sKO}*, and *DK* MuSCs after 7 days in culture (n=3 mice). Scale bar: 50 µm.

(c) Immunofluorescence-based quantification of Ki67$^+$/PAX7$^+$ MuSCs on TA muscle cryosections from *Ctrl*, *Kmt5b^{sKO}*, *p53^{sKO}*, and *DK* littermates after tamoxifen injection (n=4).
(d) Quantification of aberrant mitoses (multipolar metaphases and bridging anaphases) in cultured *Ctrl*, *Kmt5b^{sKO}*, *p53^{sKO}* and *DK* MuSCs (n=3 mice).
(e) Quantification of aneuploid MuSCs from *Ctrl*, *Kmt5b^{sKO}*, *p53^{sKO}* and *DK* mice detected by DAPI-stained metaphase spreads (n=3 mice).
(f) Quantification of *Ctrl*, *Kmt5b^{sKO}*, *p53^{sKO}*, and *DK* MuSCs with micronuclei, based on α-tubulin/DAPI immunofluorescence staining (n=3 mice).
(g) Quantification of S9.6 staining intensity (*arbitrary units* (*a.u.*)) in nuclei of cultured *Ctrl* (n=336 cells), *Kmt5b^{sKO}* (n=344 cells), *p53^{sKO}* (n=355 cells), and *DK* (n=346 cells) MuSCs. Data represent median ± interquartile ranges. n= the cell number over 3 independent experiments.
(h) Copy number variation assay (CNV-qPCR) of representative oncogenes and tumor suppressors with gain or loss of R-loops after *Kmt5b* inactivation (Fig.4f) in tumors from DK mice. *Tert* served as reference gene.
(i) PLA assay (PCNA/PolI S2P) on sections of tumors from DK mice. (n= 3 independent experiments) Scale bar: 50 µm.
(j) Quantification of PLA$^+$ cells on tumor sections per DAPI area.
(k) Simplified model illustrating the synergism of *Kmt5b* and *p53* in MuSCs for preventing formation of eRMS.
Data are shown as mean ± SEM. One-way ANOVA. Source data are provided in the Source Data file.

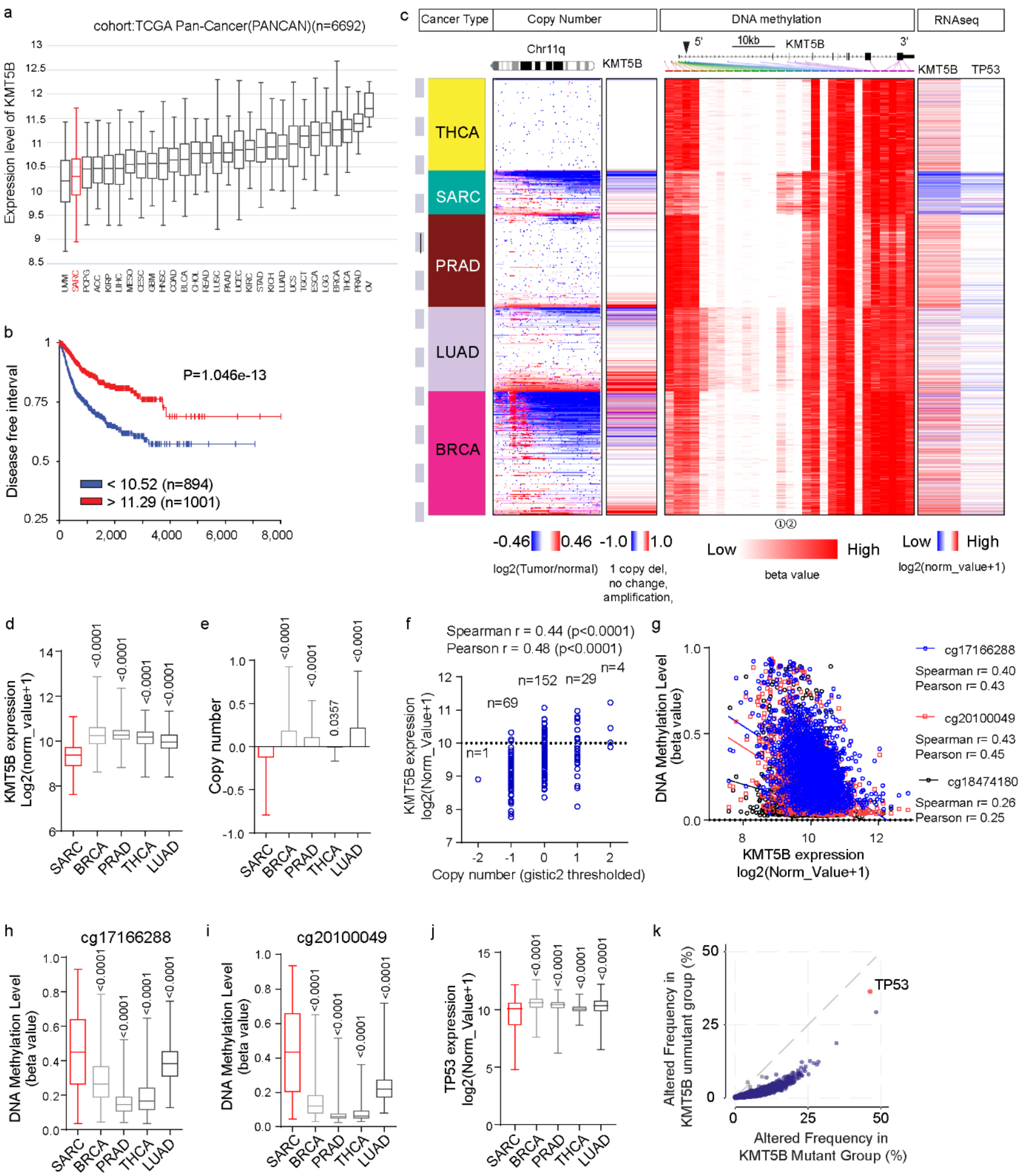

Supplementary Figure 8: Human sarcomas are characterized by low *Kmt5b* expression due to copy number loss and DNA hyper-methylation.

(a) Average expression levels of *KMT5B* in primary tumors of different TCGA Pan-Cancer types.

(b) Kaplan-Meier analysis of the disease-free interval of patients from the TCGA PANCAN project with high (n=1001) or low (n=894) levels of *KMT5B* expression. logrank test: p=1.04e-13.

(c) Xena visual spreadsheet showing genetic alterations of chr11q, gene copy number (gistic2 threshold), DNA methylation, and RNA-seq of *KMT5B* and *TP53* in primary tumors of SARC, BRCA, PRAD, THCA and LUAD from the TCGA PANCAN project. Each row corresponds to a single sample. The first column shows the scale of samples. Rows are sorted by the CNV (copy

number variation) pattern of chr11q and sub-sorted in subsequent columns. Scale bar: 100 samples. ① cg17166288, ② cg20100049. cg numbers refer to the probe numbers and relevant genomic region.
(d) *KMT5B* expression levels based on RNA-seq in primary tumors of SARC (n=250), BRCA (n=1064), PRAD (n= 488), THCA (n=495), and LUAD (n=497).
(e) Average copy number of the *KMT5B* gene locus in primary tumors of SARC (n=250), BRCA (n=1065), PRAD (n=488), THCA (n=495), and LUAD (n=497). Data are presented as mean ± SD.
(f) Correlation of *KMT5B* copy numbers (gistic2 thresholded) with *KMT5B* gene expression in TCGA SARC samples. The Spearman and Pearson correlation coefficient r is indicated. P-values indicate the significance of the correlation determined by r values and sample size.
(g) Correlation of DNA methylation levels of three *KMT5B* promoter regions with gene expression levels in 5 types of cancer. The Spearman and Pearson correlation coefficient r is indicated. P-values are (< 0.0001) and indicate the significance of the correlation determined by r values and sample size.
(h, i) DNA methylation levels of *KMT5B* promoter region, cg17166288 (h) and cg20100049 (i) in SARC (n=250), BRCA (n=764), PRAD (n=488), THCA (n=495), and LUAD (n=440).
(j) Expression of *TP53* based on RNA-seq data in SARC (n=250), BRCA (n=1064), PRAD (n=488), THCA (n=495), and LUAD (n=497).
(k) Mutations of *TP53* correlate with mutations of *KMT5B*/*KMT5B* in TCGA PANCAN.
Box plots in this figure depict the center line as median, box limits as upper and lower quartiles, and whiskers as minimum to maximum values. One-way ANOVA. Source data are provided in the Source Data file.
SARC, Sarcoma; THCA, Thyroid carcinoma; PRAD, Prostate adenocarcinoma; LUAD, Lung adenocarcinoma; BRCA, Breast invasive carcinoma

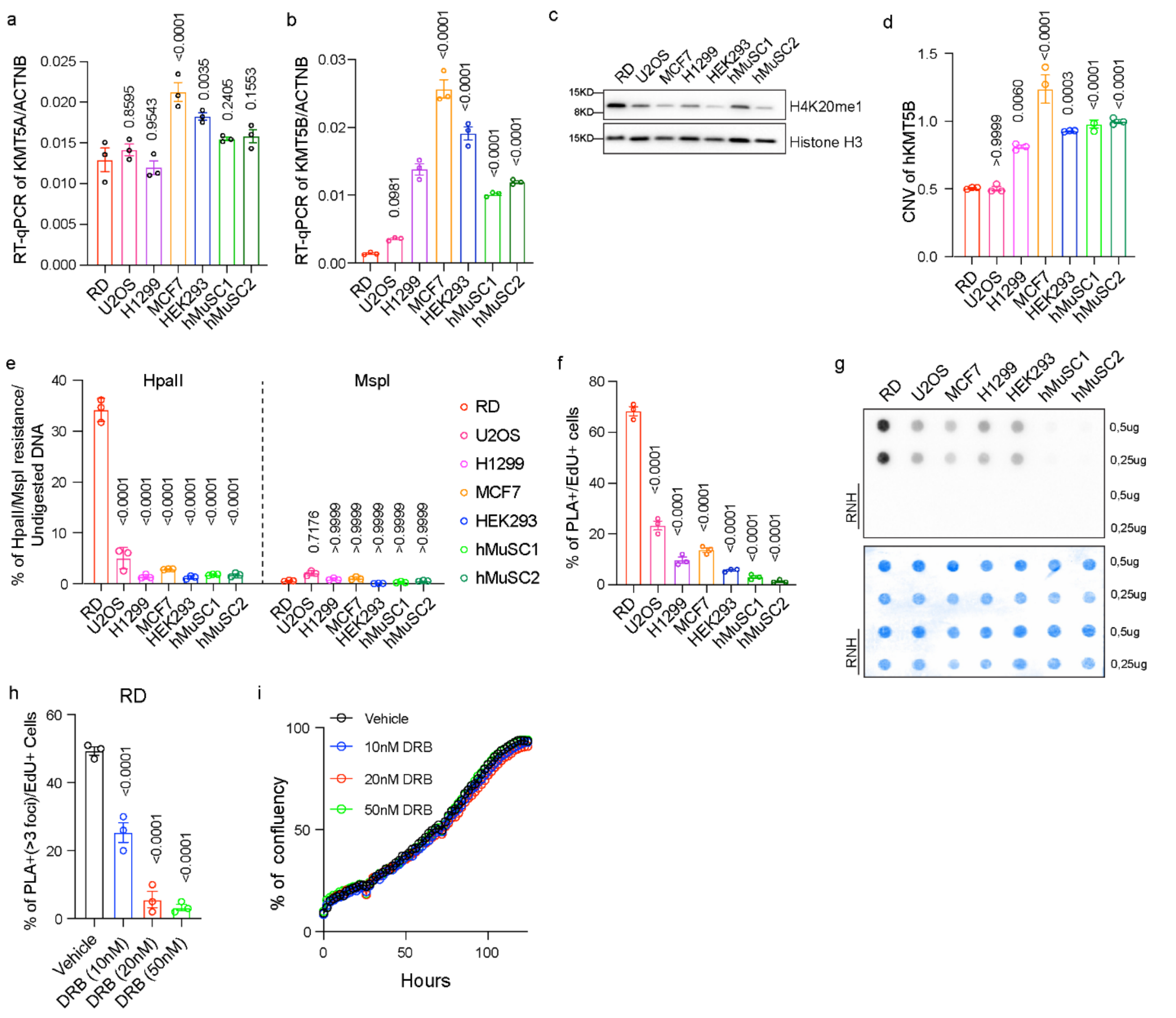

Supplementary Figure 9: Analysis of *KMT5B* expression in various cancer cell lines.
(a,b) RT-qPCR analysis of *KMT5A* (a) and *KMT5B* (b) in RD, U2OS, H1299, MCF7, HEK293, and two human myoblasts cell lines (n=3 independent experiments). *ACTN* served as the reference gene.
(c) Western blot analysis of H4K20me1 in RD, U2OS, H1299, MCF7, HEK293, and two human myoblasts cell lines. Histone H3 served as the internal control.
(d) Copy number variation (CNV) assay of *KMT5B* in RD, U2OS, H1299, MCF7, HEK293, and two human myoblasts cell lines (n=3 independent experiments). *RPR1* served as the reference gene.
(e) Methylation status of the h*KMT5B* gene promoter accessed by HpaII/MspI-qPCR analysis (n=3 independent experiments).
(f) Quantification of PLA (PCNA:PolII S2P)$^{+}$/EdU^{+} cells in different cancer and normal human cell lines (n=3 independent experiments).
(g) Dot blot analysis of R-loops by S9.6 antibody using genomic DNA from different cancer and normal human cell lines.
(h) Quantification of PLA (PCNA:PolII S2P)$^{+}$/EdU^{+} cells in vehicle and different concentration of DRB (10, 20, 50nM) treated RB cells (n=3 independent experiments).
(i) Proliferation assay of RB cells treated with vehicle and different concentration of DRB (10, 20, 50nM).

Data are shown as mean ± SEM. One-way ANOVA. Source data are provided in the Source Data file.

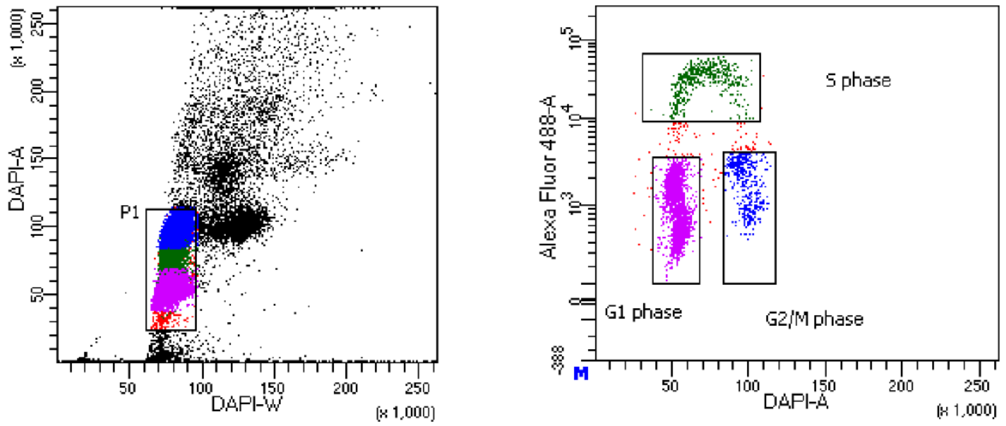

Supplementary Figure 10: Detailed gating strategy for G0/G1, S, and G2/M phases using CD FACS Diva v8 software.
