# Supplementary material for: Replication collisions induced by de-repressed S-phase transcription are connected with malignant transformation of adult stem cells
Source: Nat Commun. 2022 Nov 14;13:6907. doi: 10.1038/s41467-022-34577-y (PMC9663592; doi:10.1038/s41467-022-34577-y)
Supplement: Supplementary file 3 — Description of Additional Supplementary Files [file 41467_2022_34577_MOESM3_ESM.pdf]

Description of Additional Supplementary Files

File Name: Supplementary Data 1
Description: Primers for genotyping ,RT-qPCR,copy number variation assay and DNA methylation assay.

File Name: Supplementary Data 2
Description: Clinical Data of Cancer Patients Related to Supplementary Figure 8.

File Name: Supplementary Data 3
Description: Probes and genomic locations for 450K methylation array shown in Supplementary Figure 8c.
