# Supplementary material for: Replication collisions induced by de-repressed S-phase transcription are connected with malignant transformation of adult stem cells
Source: Nat Commun. 2022 Nov 14;13:6907. doi: 10.1038/s41467-022-34577-y (PMC9663592; doi:10.1038/s41467-022-34577-y)
Supplement: Supplementary file 7 — Reporting Summary [file 41467_2022_34577_MOESM7_ESM.pdf]

nature portfolio

Corresponding author(s): Thomas Braun; Xuejun Yuan

Last updated by author(s): Oct 15, 2022

Reporting Summary

Nature Portfolio wishes to improve the reproducibility of the work that we publish. This form provides structure for consistency and transparency in reporting. For further information on Nature Portfolio policies, see our Editorial Policies and the Editorial Policy Checklist.

Statistics

For all statistical analyses, confirm that the following items are present in the figure legend, table legend, main text, or Methods section.

n/a	Confirmed	
☐	☒	The exact sample size (*n*) for each experimental group/condition, given as a discrete number and unit of measurement
☐	☒	A statement on whether measurements were taken from distinct samples or whether the same sample was measured repeatedly
☐	☒	The statistical test(s) used AND whether they are one- or two-sided<br>*Only common tests should be described solely by name; describe more complex techniques in the Methods section.*
☐	☒	A description of all covariates tested
☐	☒	A description of any assumptions or corrections, such as tests of normality and adjustment for multiple comparisons
☐	☒	A full description of the statistical parameters including central tendency (e.g. means) or other basic estimates (e.g. regression coefficient) AND variation (e.g. standard deviation) or associated estimates of uncertainty (e.g. confidence intervals)
☐	☒	For null hypothesis testing, the test statistic (e.g. *F*, *t*, *r*) with confidence intervals, effect sizes, degrees of freedom and *P* value noted<br>*Give P values as exact values whenever suitable.*
☒	☐	For Bayesian analysis, information on the choice of priors and Markov chain Monte Carlo settings
☒	☐	For hierarchical and complex designs, identification of the appropriate level for tests and full reporting of outcomes
☒	☐	Estimates of effect sizes (e.g. Cohen's *d*, Pearson's *r*), indicating how they were calculated

Our web collection on statistics for biologists contains articles on many of the points above.

Software and code

Policy information about availability of computer code

Data collection	Zeiss Zi-Imager-software Zen2 6.1.7601<br>Leica Sp8-Software Las X 3.5.7.23225<br>FACS AriaTM III(BD Biosciences)-BD FACSDiva™ v8 Software
Data analysis	R language 3.4.1<br>FastQC 0.11.8<br>STAR 2.7.3a;2.6.1d<br>Picard 2.21.7; 2.18.16<br>Trimmomatic version 0.39<br>BEDTools 2.29<br>DeepTools2<br>BioVenn 1.1.1<br>pyGenomeTracks<br>Epic2 0.0.41<br>UROPA<br>NRSA v2<br>DAVID Bioinformatics Resources 6.8<br>GraphPad Prism 8<br>Galaxy platform<br>FlowJo<br>SonoLab 7.3<br>Fiji

Cut&Run
Sequencing was performed on the NextSeq500 instrument (Illumina) using v2 chemistry, resulting in minimum of 28M reads per library with 1x75bp pair end setup.
Raw reads were assessed for quality, adapter content and duplication rates with FastQC 0.11.8 (Andrews S. 2010, FastQC: a quality control tool for high throughput sequence data. Available online at: http://www.bioinformatics.babraham.ac.uk/projects/fastqc). Trimmomatic version 0.39 was employed to trim reads after a quality drop below a mean of Q15 in a window of 5 nucleotides (Bolger et al., 2014). Only reads longer than 15 nucleotides were cleared for further analyses. Trimmed and filtered reads were aligned versus the Ensembl mouse assembly mm10 (GRCm38 release 99) using STAR 2.7.3a retaining only unique alignments (Dobin et al., 2013). Reads were deduplicated using Picard 2.21.7 (http://broadinstitute.github.io/picard/) when working with next generation sequencing data in BAM format to mitigate PCR artefacts leading to multiple copies of the same original fragment. Count matrices was produced similarly based on the Spike-in normalized coverage of all mouse genes per sample. All genes located on chromosomes X or Y were excluded from the analysis. Differential genes were identified with a normalized count > 50, log2 fold change < -0.5 or >0.5, and multiple testing adjusted P-Value (padj) < 0.1 as determined by DEseq2 (Anders and Huber, 2010).
Venn diagram was generated using BioVenn 1.1.1 (R package)(Hulsen et al., 2008). Identified DEGs were uploaded to the online software DAVID for KEGG pathway analyses (Huang da et al., 2009a, b). Coverage heatmaps and profile plots are based on Spike-in normalized BigWig files produced using Deeptools were generated by deepTool2 (Ramirez et al., 2016). Genomic tracks of sequencing data were visualized using pyGenomeTracks (Lopez-Delisle et al., 2020).

DRIP-seq
Raw data were processed similarly to the CUT&RUN data. Peaks were called comparing the respective treatment to Input samples using Epic2 0.0.41(Patten et al., 2018). Peaks overlapping ENCODE blacklisted regions (known misassemblies, satellite repeats) were excluded. To enable comparison of peaks in different samples, the resulting lists of significant peaks were overlapped and unified. Sample counts for union peaks were produced using bigWigAverageOverBed (UCSC Toolkit). Reads mapping against Spike-in organism Drosophila melanogaster (BDGP6) were counted for each sample and used to normalize the matrix of union peak scores. Union peaks were annotated with the gene having the longest overlap based on reference data of Ensembl release 99. All peaks located on chromosomes X or Y were excluded from the analysis. Differential peaks/genes were identified with a normalized count > 30, fold change < -1.5 or >1.5, and multiple testing adjusted P-Value (padj) < 0.1 as determined by DEseq2. Peaks were annotated with UROPA(Kondili et al., 2017) based on the overlap of the peak center using the following priority of feature types: 1) promoter (TSS +- 1kb), 2) TES region (TES + 3kb downstream), 3) gene, 4) intergenic. If multiple genes of the same feature type were intersected, the one with the longest overlap was selected. The genomic background represents the distribution of features if reads were randomly placed.

PRO-seq
Raw reads were assessed for quality, adapter content and duplication rates with FastQC 0.11.8 (Andrews S. 2010, FastQC: a quality control tool for high throughput sequence data. Available online at: http://www.bioinformatics.babraham.ac.uk/projects/fastqc). Trimmomatic version 0.39 was employed to trim reads after a quality drop below a mean of Q15 in a window of 5 nucleotides (Bolger et al., 2014). Only reads longer than 15 nucleotides were cleared for further analyses. Trimmed and filtered reads were aligned versus the Ensembl mouse assembly mm10 (GRCm38 release vM15) using STAR 2.6.1d retaining one random alignment in case of multi-mapping reads (Dobin et al., 2013). Reads were deduplicated using Picard 2.18.16 (http://broadinstitute.github.io/picard/) when working with next generation sequencing data in the BAM format to mitigate PCR artefacts leading to multiple copies of the same original fragment.. Target features were produced by extracting all isoforms of protein-coding genes of Ensembl GRCm38 release vM15 with length >= 1000 nt and creating for each: 1) TSS region (start=TSS, stop=400 nt downstream of TSS), 2) body region (start=400 nt downstream of TSS, stop=TES). Read counts for TSS/body features were created using bigWigAverageOverBed (UCSC Toolkit) by only counting reads from the correct strand. Count matrices for TSS/body features were TPM normalized (sequencing depth, feature length) and combined. All isoforms with an average TPM < 0.5 in either TSS or body features were removed. One isoform was selected for each gene as the primary transcript based on the largest average TPM score on the TSS feature. The pausing index ratio (PI) was computed as TPM TSS / TPM Genebody. Sequencing depth normalized BigWig files were produced using Bedtools (Quinlan and Hall, 2010) and wigToBigWig (UCSC Toolkit). eRNAs were predicted using the NRSA v2 pipeline which locates nascent bidirectional transcripts (Wang et al., 2018). Volcano plot was created in R with ggplot2 package. All eRNAs/genes located on chromosomes X or Y were excluded from the analysis. eRNAs (fold change>0.5 or <-0.5 and FDR<0.1) was colored with significant change. Coverage heatmaps and profile plots are produced using Deeptools were generated by deepTool2 (Ramirez et al., 2016). Genomic tracks of sequencing data were visualized using pyGenomeTracks (Lopez-Delisle et al., 2020).

For manuscripts utilizing custom algorithms or software that are central to the research but not yet described in published literature, software must be made available to editors and reviewers. We strongly encourage code deposition in a community repository (e.g. GitHub). See the Nature Portfolio guidelines for submitting code & software for further information.

Data

Policy information about availability of data

All manuscripts must include a data availability statement. This statement should provide the following information, where applicable:

- Accession codes, unique identifiers, or web links for publicly available datasets
- A description of any restrictions on data availability
- For clinical datasets or third party data, please ensure that the statement adheres to our policy

BioProject: PRJNA412267 publicly available datasets Fig.3b,c
GEO accession number: GSE173257 (https://www.ncbi.nlm.nih.gov/geo/query/acc.cgi?acc=GSE173257) Fig. 3a-3c; Extended Data Fig.4b-e
GEO accession number: GSE173258 (https://www.ncbi.nlm.nih.gov/geo/query/acc.cgi?acc=GSE173258) Fig. 6a-6i ; Fig. S6a-d
GEO accession number: GSE173259 (https://www.ncbi.nlm.nih.gov/geo/query/acc.cgi?acc=GSE173259) Fig.2c,2d 2f-2k; Fig. 6c; Extended Data Fig.3d-g
All other data generated during the current study are included in the Source Data file/ Supplementary Information, which are provided together with this paper.

Field-specific reporting

Please select the one below that is the best fit for your research. If you are not sure, read the appropriate sections before making your selection.

☒ Life sciences ☐ Behavioural & social sciences ☐ Ecological, evolutionary & environmental sciences

For a reference copy of the document with all sections, see nature.com/documents/nr-reporting-summary-flat.pdf

Life sciences study design

All studies must disclose on these points even when the disclosure is negative.

Sample size	Sample size were determined based on established practice and applicable standards. We opted for sample sizes which are commonly used sample sizes in the field.<br>For in vivo studies, a minimum of three biological replicates were analyzed. For In vitro studies, experiments in which data were not quantified were performed with at least two replicates. Each experiment in which data were quantified was performed with at least 3 replicates.
Data exclusions	No data were excluded.
Replication	All in vivo studies were performed once with indicated number of animals. All other experiments have been repeated with similar results at least three times. Sample sizes and statistical analyses and significance levels are all indicated in the figure legends or the method part.
Randomization	All animals were numbered and experiments were performed in a blinded pattern. After data collection, genotypes were revealed and animals assigned to groups for analysis.
Blinding	In vivo experiments were performed in a blinded pattern. For in vitro experiments, the researchers were not blinded during data collection and analysis. However, the experiments are repeated by independent researchers to validate the results. Positive controls, negative controls and samples were analyzed in exactly the same manner.

Reporting for specific materials, systems and methods

We require information from authors about some types of materials, experimental systems and methods used in many studies. Here, indicate whether each material, system or method listed is relevant to your study. If you are not sure if a list item applies to your research, read the appropriate section before selecting a response.

Materials & experimental systems

n/a	Involved in the study	
☐	☒	Antibodies
☐	☒	Eukaryotic cell lines
☒	☐	Palaeontology and archaeology
☐	☒	Animals and other organisms
☒	☐	Human research participants
☒	☐	Clinical data
☒	☐	Dual use research of concern

Methods

n/a	Involved in the study	
☐	☒	ChIP-seq
☐	☒	Flow cytometry
☒	☐	MRI-based neuroimaging

Antibodies

Antibodies used	Anti-γH2AX rabbit polyclonal antibody, Cell Signaling Cat# 2577, IF,WB(1:1000)<br>Anti-53BP1 rabbit polyclonal antibody, Abcam Cat# ab36823, IF (1:1000)<br>Anti-P53 mouse monoclonal antibody [PAb 240], Abcam Cat# ab26, WB(1:1000)<br>Anti-α-tubulin mouse monoclonal antibody (clone B-5-1-2), Sigma-Aldrich Cat# T6074, IF (1:2000)<br>Anti-centrin-2 rabbit polyclonal antibody, Merck Millipore Ca# ABE480, IF (1:1000)<br>Anti-pan-actin rabbit polyclonal antibody,Cell Signaling Cat# 4968, WB(1:1000)<br>Anti-Ki67 rabbit polyclonal antibody, Abcam Cat# ab15580, IF (1:1000)<br>Anti-H4K20me1 rabbit polyclonal antibody, Abcam Cat# ab9051, IF(1:2000) Cut&Run(1:100)<br>Anti-H4K20me2 mouse monoclonal antibody, Diagenode Cat# C15200205, IF(1:2000)<br>Anti-H4K20me3 rabbit polyclonal antibody, Abcam Cat# ab9053, IF(1:2000)<br>Anti-histone H3 rabbit polyclonal antibody, Abcam Cat# ab18521, WB(1:2000)<br>Anti- RNA polymerase II Ser2P rabbit polyclonal antibody, Abcam Cat# ab5095, WB,IF(1:1000) Cut&Run(1:100)<br>Anti- RNA polymerase II Ser5P mouse monoclonal antibody (3E8), Active motif Cat# 61085, WB(1:1000)<br>Anti-RNA polymerase II (CTD4H8) mouse monoclonal antibody, Santa Cruz Cat# sc-47701, WB(1:1000)<br>Anti-DNA-RNA Hybrid [S9.6] mouse monoclonal antibody, Kerafast Cat# ENH001, Dot blot,IF(1:1000)<br>Anti-PCNA mouse monoclonal antibody(PC10), Abcam Cat# ab29, IF(1:1000)<br>Anti-phospho-RPA32 (Ser33) rabbit polyclonal antibody, Bethyl Cat# A300-246A-M, IF(1:1000)<br>Anti-phospho-RPA32 (S4/S8) rabbit polyclonal antibody, Bethyl Cat# A300-245A-M, IF,WB(1:1000)

Anti-phospho-RPA32 (Thr21) rabbit polyclonal antibody, GeneTex Cat# GTX130432, WB(1:1000)
Anti-RPA32 rabbit polyclonal antibody, Thermo Fisher Cat# PA5-22256, WB(1:1000)
Anti-phospho-ATR rabbit polyclonal antibody, Cell Signaling Cat# 2853, IF(1:1000)
Anti-laminin rabbit polyclonal antibody, Abcam Cat# ab11575, IF(1:2000)
Anti-MYOD1 rabbit polyclonal antibody, Abcam Cat# ab64159, IF (1:1000)
Anti-myogenin mouse monoclonal antibody (F5D), BD Bioscience Cat# 556358, IF (1:1000)
Anti-PAX7 mouse monoclonal antibody (Pax7), R&D System Cat# MAB1675, IF (1:1000)
Anti-desmin rabbit polyclonal antibody, Sigma Aldrich Cat# D8281, IF (1:1000)
Rabbit IgG, Diagenode Cat# C15410206, Cut&Run (1:100)
Anti-V5 Tag Mouse monoclonal antibody, ThermoFisher Cat# R960-25,WB (1:1000)
Anti-HA tag Rabbit polyclonal antibody,Abcam Cat# ab9110,WB (1:1000)
Anti-SETD8 Rabbit polyclonal antibody, Millipore Cat#06-1304,WB(1:1000)
Anti-PHF8 Rabbit polyclonal antibody, Bethyl Laboratories Catalog # A301-772A,WB(1:1000)
Goat anti-mouse HRP,Millipore Cat#12-349, WB(1:10000)
Anti-mouse IgG Alexa Fluor 488, Invitrogen Cat#A11001,IF (1:500)
Anti-rat IgG Alexa Fluor 594, Invitrogen Cat#A11007,IF (1:500)
Rat anti-bromodeoxyuridine(BrdU) (BU1/75, Acris 1:1,000)
Mouse anti-bromodeoxyuridine (BrdU) (B44, Becton Dickinson 1:500)

Validation

Anti-γH2AX antibody (Cell Signaling 2595) was validated by immunofluorescent staining (IF) in UV-treated or untreated 293 cells.
Anti-53BP1 (Abcam ab36823) antibody was validated by western blot (WB) in mouse MEF, mouse pancreatic cancer cells, Hela cells and U2OS cells.
Anti-P53 antibody (Abcam ab26) was validated by immunoprecipitation (IP) in Hela and HCT116 cells and by WB in mouse tissues and ES cells.
Anti- -α-tubulin(Sigma-Aldrich T6074) was validated by immunocytochemistry (ICC) using cultured chicken fibroblasts (CFB).
Anti-Centrin-2 (ABE480) was validated by ICC in MDCK cells in an independent laboratory (Koch, A., et al. (2010). Mol Biol Cell. 21(2):219-231.).
Anti-pan-actin (cell signaling #4968) was analyzed by WB of extracts from HeLa, L929, C6 and COS cells. Anti-Ki67 antibody (ab15580) was validated extensively by ICH in different human and mouse tissues and cell lines.
Anti-H4K20me1/H4K20me2/H4k20me3 were validated in the lab by dot blot using specific peptide. Anti-H4K20me1 (ab9051) was validated by WB, Flow Cyt, ICC/IF, ChIP, ChIP-sequencing in different human and mouse cells. Anti-H4K20me2 (ab9052) was validated by WB and IF in Hela, multiple myeloma cells, and mouse ES cells. Anti-H4K20me3 (ab9053) was validated by ICC in human and mouse tissue samples.
Anti-Histone H3 antibody (ab18521) was validated by ICC in HeLa cells and by WB in calf thymus and HeLa cells.
Anti-RNA Pol II S2P antibody (ab5095) was validated by ChIP-seq in HEK293 cells and AML cells (https://doi.org/10.1038/s41586-019-1618-0; https://doi.org/10.1038/s41586-019-1842-7) and by WB in U2OS, CHO, BHK-21 and mouse MEF cells. Anti-RNA PolII S5P antibody was validated by WB in Hela cells and IF in U2OS cells. Pol II Antibody (CTD4H8): sc-47701. Western blot analysis of Pol II expression in A431, 3T3-L1, A673, U2OS and AT3B-1 whole cell lysates.
S9.6 antibody: Affinity Binding Assay: Clone S9.6 bound the DNA-RNA heteropolymer and poly(I)-poly(dC) equally, but 100-fold higher levels of poly(A)-poly(dT) were required to achieve a similar degree of binding. Single-stranded DNA, double-stranded DNA and RNA, and ribosomal RNA were not bound by clone S9.6 (Boguslawski, S.J., et al. (1986). J. Immunol Methods. 89(1):123-130). ICC and DRIP-seq: HeLa cells (Bhatia, V., et al. (2014). Nature. 511(7509):362-365) and budding yeast (El Hage, A., et al. (2014). PLoS Genet. 10(10):e1004716).
Anti-PCNA (ab29) was validated by WB in HeLa, HEK293, A431 whole cell lysates, PC12, SV40LT-SMC, NIH 3T3, rat liver and rat heart and by IHC-Fr in mouse embryonic brain and HeLa.
Anti-phospho-RPA32 (Ser33) antibody (Bethyl Cat# A300-246A-M), Anti-phospho-RPA32 (S4/S8) antibody (Bethyl Cat# A300-245A-M) and Anti-RPA32 antibody were validated by WB in HeLa cells treated with etoposide or mock treated cells.
Anti-phospho-RPA32 (Thr21) antibody (GeneTex Cat# GTX130432) was validated in etoposide treated 293T cells.
Anti-phospho-ATR antibody (Cell Signaling Cat# 2853) was validated by WB in Raw264.7, SV-T2 and HT-29 cells that were untreated or UV-treated.
Anti-laminin antibody (ab11575) was validated by IF in mouse brain tissue.
Anti-MYOD1 antibody (ab64159) was validated by WB in mouse muscle tissue.
Anti-myogenin antibody (BD #556358) was validated by WB and IF in human rhabdomyosarcoma cells RH30.
Anti-Pax7 antibody (R&D Systems MAB1675) was validated by ICC in C2C12 Mouse Cell Line.
Anti-desmin antibody (Sigma Aldrich # D8281) was validated by immunohistochemistry of zebrafish intestines sections and immunodetection in fish embryo samples and immunolabeling of myofibers.
Anti-V5 Tag antibody (R960-25) is functionally tested against 20 ng of an E. coli expressed fusion protein containing a V5 epitope using a chemiluminescent substrate at a 1 minute exposure. This antibody has also been tested in Western blot against 25 ng of recombit Positope™ protein.
Anti-HA tag antibody (ab9110) was validated by WB in293FT cells transfected with 15kDa HA tagged Vpr (an HIV1 accessory protein)
Anti-SETD8 antibody (06-1304) was validated by western blot in C2C12 cells and HeLa cells.
Anti-PHF8 antibody (A301-772A) was validated by western blot using whole cell lysate (15 and 50 µg) from Jurkat, HeLa, HEK293T, and NIH 3T3 cells.
Goat anti-mouse HRP(12-349) was validated by western blot in different mouse tissue and cell lines.
Anti-mouse IgG Alexa Fluor 488 (A11001) was validated by immunofluorescent staining in mouse muscle sections and human cancer cell lines.
Anti-rat IgG Alexa Fluor 594 (A11007) was validated by immunofluorescent staining in mouse muscle sections and human cancer cell lines
Rat anti-BrdU) (BU1/75) and mouse anti-BrdU) (B44) were validated by DNA fiber assay with the cells pulsed with only IdU and CIdU.

Eukaryotic cell lines

Policy information about cell lines

Cell line source(s)	RD source ATCC #CCL-136<br>U-2 OS ATCC #HTB-96<br>MCF7 ATCC #HTB-22<br>H1299 ATCC CRL-5803<br>HEK293 ATCC CRL-1573<br>Platinum-E (Plat-E) Cellbiolabs RRID:CVCL_B488<br>human myoblast cell lines were provided by the platform for immortalization of human cells of the institut de Myologie(Paris, France). The initial biopsies, from which the cell lines were generated, were provided by MyoBank, the tissue bank of the institut de Myologie in Paris, affiliated with EuroBioBank. MyoBank has received approval from the French Ministry of Higher Education, Research and Innovation to distribute human samples for research (Authorization code AC-2019-3502).
Authentication	Each cell line used was morphologically confirmed according to the information provided by the culture collections.
Mycoplasma contamination	Tested for being Mycoplasma free
Commonly misidentified lines<br>(See ICLAC register)	No Commonly misidentified lines were used

Animals and other organisms

Policy information about studies involving animals; ARRIVE guidelines recommended for reporting animal research

Laboratory animals	The following mouse strains were used in this study:<br>Ctrl Pax7CE+/p // Kmt5b+/+// p53+/+<br>Kmt5bsKO Pax7CE+/p // Kmt5bfl/fl // p53+/+<br>p53sKO Pax7CE+/p // p53fl/fl // Kmt5b+/+<br>DK Pax7CE+/p // Kmt5bfl/fl // p53fl/fl<br>All mice used in this study were drug and test naive, healthy prior to the studies, not used in previous procedures and maintained in individually ventilated cages, at 22.5 °C ± 1 °C and a relative humidity of 50% ± 5% with controlled illumination (12 h dark/light cycle). Mice were given ad libitum access to food and water. All mouse strains were backcrossed and maintained on a C57BL/6 genetic background. Female and male animals at the age between 8 to 12 weeks to equal proportions were analyzed in this study. None of the determined parameters in this study correlated with animal sex.
Wild animals	Studies did not involve wild animals.
Field-collected samples	Studies did not involve samples collected in the field.
Ethics oversight	All animal experiments were done in accordance with the Guide for the Care and Use of Laboratory Animals published by the US National Institutes of Health (NIH Publication No. 85-23, revised 1996) and were approved by Animal Rights Protection Committee of the State of Hessen (Regierungspraesidium Darmstadt, Wilhelminenstr. 1-3, 64283 Darmstadt, Germany) with the project number B2/1137.

Note that full information on the approval of the study protocol must also be provided in the manuscript.

ChIP-seq

Data deposition

☒ Confirm that both raw and final processed data have been deposited in a public database such as GEO.

☒ Confirm that you have deposited or provided access to graph files (e.g. BED files) for the called peaks.

Data access links<br>*May remain private before publication.*	https://www.ncbi.nlm.nih.gov/geo/query/acc.cgi?acc=GSE173335
Files in database submission	GSM5264398 PROseq_WT_1<br>GSM5264399 PROseq_WT_2<br>GSM5264400 PROseq_WT_3<br>GSM5264401 PROseq_SH1sKO_1<br>GSM5264402 PROseq_SH1sKO_2<br>GSM5264403 PROseq_SH1sKO_3<br>GSM5264404 DRIPseq_Input_WT_1<br>GSM5264405 DRIPseq_Input_SH1sKO_1<br>GSM5264406 DRIPseq_WT_1<br>GSM5264407 DRIPseq_WT_2<br>GSM5264408 DRIPseq_WT_3<br>GSM5264409 DRIPseq_SH1sKO_1

GSM5264410 DRIPseq_SH1sKO_2
GSM5264411 DRIPseq_SH1sKO_3
GSM5264412 CnR_IGG_WT_1
GSM5264413 CnR_IGG_SH1sKO_1
GSM5264414 CnR_H4K20me1_WT_1
GSM5264415 CnR_H4K20me1_WT_2
GSM5264416 CnR_H4K20me1_SH1sKO_1
GSM5264417 CnR_H4K20me1_SH1sKO_2
GSM5264418 CnR_PolIIS2P_WT_1
GSM5264419 CnR_PolIIS2P_WT_2
GSM5264420 CnR_PolIIS2P_SH1sKO_1
GSM5264421 CnR_PolIIS2P_SH1sKO_2

Genome browser session
(e.g. UCSC)

N/A

Methodology

Replicates

Two biological replicates for Cut&Run H4K20me1 and PolIIS2P; three biological replicates for PROseq and DRIPseq

Sequencing depth

total read unique mapped reads
GSM5264398 PROseq_WT_1 40360048 30094023 paired end
GSM5264399 PROseq_WT_2 24883263 17354023 paired end
GSM5264400 PROseq_WT_3 32673450 27072787 paired end
GSM5264401 PROseq_SH1sKO_1 53276283 40485430 paired end
GSM5264402 PROseq_SH1sKO_2 41160868 29833654 paired end
GSM5264403 PROseq_SH1sKO_3 47202239 36277455 paired end
GSM5264404 DRIPseq_Input_WT_1 31186470 25375257 paired end
GSM5264405 DRIPseq_Input_SH1sKO_1 36175268 29439277 paired end
GSM5264406 DRIPseq_WT_1 31556264 24162188 paired end
GSM5264407 DRIPseq_WT_2 29501899 22475984 paired end
GSM5264408 DRIPseq_WT_3 42576313 31377556 paired end
GSM5264409 DRIPseq_SH1sKO_1 23315825 15827197 paired end
GSM5264410 DRIPseq_SH1sKO_2 26689789 17982880 paired end
GSM5264411 DRIPseq_SH1sKO_3 29775444 20237033 paired end
GSM5264412 CnR_IGG_WT_1 63930285 40693849 paired end
GSM5264413 CnR_IGG_SH1sKO_1 41939352 26316666 paired end
GSM5264414 CnR_H4K20me1_WT_1 22498985 19251194 paired end
GSM5264415 CnR_H4K20me1_WT_2 23096057 19816557 paired end
GSM5264416 CnR_H4K20me1_SH1sKO_1 36177644 31778368 paired end
GSM5264417 CnR_H4K20me1_SH1sKO_2 25840700 22588835 paired end
GSM5264418 CnR_PolIIS2P_WT_1 36514481 26617481 paired end
GSM5264419 CnR_PolIIS2P_WT_2 37174408 29557932 paired end
GSM5264420 CnR_PolIIS2P_SH1sKO_1 44971340 35035155 paired end
GSM5264421 CnR_PolIIS2P_SH1sKO_2 44733667 34359095 paired end

Antibodies

Anti- RNA polymerase II Ser2P rabbit polyclonal antibody, Abcam Cat# ab5095
Anti-H4K20me1 rabbit polyclonal antibody, Abcam Cat# ab9051
Anti-DNA-RNA Hybrid [S9.6] mouse monoclonal antibody, Kerafast Cat# ENH001

Peak calling parameters

DRIPseq Peaks were called comparing the respective treatment to Input samples using Epic2 0.0.41(Patten et al., 2018). Peaks overlapping ENCODE blacklisted regions (known misassemblies, satellite repeats) were excluded. To enable comparison of peaks in different samples, the resulting lists of significant peaks were overlapped and unified. Sample counts for union peaks were produced using bigWigAverageOverBed (UCSC Toolkit). Reads mapping against Spike-in organism Drosophila melanogaster (BDGP6) were counted for each sample and used to normalize the matrix of union peak scores. Union peaks were annotated with the gene having the longest overlap based on reference data of Ensembl release 99. All peaks located on chromosomes X or Y were excluded from the analysis. Differential peaks/genes were identified with a normalized count > 30, fold change < -1.5 or >1.5, and multiple testing adjusted P-Value (padj) < 0.1 as determined by DEseq2.

Data quality

mode: epic2; filter: FDR <= 0.05, minimum treatment reads >= 1, enrichment >= 1, log10(qvalue) <= -0.5; not overlapping blacklisted regions like satellite repeats or misassemblies (ENCODE) reproducible = at least two samples / group must show a similar normalized and background-corrected expression (absolute log2fc <= 0.585)

Software

FastQC 0.11.8 (https://www.bioinformatics.babraham.ac.uk/projects/fastqc/)
STAR 2.7.3a
Picard 2.21.7 (https://broadinstitute.github.io/picard/)
Trimmomatic version 0.39
BEDTools
DeepTools2
Epic2 0.0.41
UROPA
NRSA v2

Flow Cytometry

Plots

Confirm that:

☒ The axis labels state the marker and fluorochrome used (e.g. CD4-FITC).

☒ The axis scales are clearly visible. Include numbers along axes only for bottom left plot of group (a 'group' is an analysis of identical markers).

☒ All plots are contour plots with outliers or pseudocolor plots.

☒ A numerical value for number of cells or percentage (with statistics) is provided.

Methodology

Sample preparation	Cells were pulse labeled with 10 µM EdU for 30 min before fixation. EdU incorporation was visualized using Click-iT™ EdU Alexa Fluor™ 488 Flow Cytometry Assay (Invitrogen) according to the manufacturer's protocol and followed by DAPI staining on ice for more than 1 hours. FACS analysis was perform on BD LSRFortessa™ Cell Analyzer and data was analyzed using FlowJo software. Fixed S phase and G1 phase cells were sorted using BD FACSAria™ III Cell Sorter.
Instrument	BD LSRFortessa™ Cell Analyzer; BD FACSAria™ III Cell Sorter
Software	BD FACSDiva; FlowJo,LLC
Cell population abundance	Sorted cells were reanalyzed to assess purity,>90% purity was achieved.
Gating strategy	FSC-A versus SSC-A plot to discriminate cells versus debris. DAPI-H versus DAPI-A plot to discriminate singlets versus doublets. DAPI-A versus EdU-488 -A to gate for individual cell cycle phase. S phase population was defines by comparing to FMO (Fluorescence Minus One) EdU-488.

☒ Tick this box to confirm that a figure exemplifying the gating strategy is provided in the Supplementary Information.
